# Patient-reported satisfaction after ultrasound-assisted liposuction with infra-areolar excision for gynecomastia: a retrospective cross-sectional from Palestine

**DOI:** 10.1186/s13104-026-07735-4

**Published:** 2026-02-25

**Authors:** Mahdi Aljamal, Ali Shakhshir, Amro J. A. Sulaiman, Mohammed A. Zeidalkilani

**Affiliations:** 1 Department of Surgery, Faculty of Medicine and Health Sciences, Arab American University, Jenin, Palestine; 2 Present Address: Department of Medicine, Faculty of Medicine and Health Sciences, Arab American University, Jenin, Palestine; 3 Faculty of Medicine and Health Sciences, Arab American University, Jenin, Palestine

**Keywords:** Gynecomastia, Ultrasound-assisted liposuction (UAL), Glandularexcision, Satisfaction, Body-Q, Cross-sectional retrospective study, Palestine

## Abstract

**Background:**

Gynecomastia can have significant psychosocial impacts. Ultrasound-assisted liposuction (UAL) combined with glandular excision has emerged as a preferred technique for contour correction with minimal scarring. This study evaluates surgical outcomes and patient satisfaction following UAL with glandular excision.

**Methodology:**

This was a retrospective cross-sectional study of male patients who underwent bilateral gynecomastia correction using ultrasound-assisted liposuction with infra-areolar excision between September 2022 and June 2024 in Palestine. Clinical and perioperative variables were extracted from medical records, and outcomes were supplemented with patient-reported satisfaction using the validated Arabic BODY-Q chest and nipple modules collected via an online follow-up questionnaire. Nonparametric group comparisons, Spearman correlation, and multivariable linear regression were used to identify predictors of satisfaction (SPSS v21).

**Results:**

A total of 133 patients were included (median age 28 years; median BMI 26 kg/m²). Most cases were physiological and primarily Simon grade IIa/IIb. The median aspirated fat volume was 350 Ml. Postoperative complications were generally infrequent; the most common were asymmetry, redundant skin, hematoma, and contour irregularity, and 12% of patients underwent revision surgery. Overall satisfaction was high in both BODY-Q domains. Chest satisfaction decreased with increasing gynecomastia grade and was independently lower in patients with contour irregularity, undesirable scars, redundant skin, and recurrence. Nipple satisfaction was independently lower with contour irregularity, undesirable scars, redundant skin, and nipple–areola complex adherence. Chest and nipple satisfaction scores were positively correlated.

**Conclusion:**

Ultrasound-assisted liposuction with infra-areolar excision achieved high patient-reported satisfaction with a low-to-moderate complication profile in this Palestinian cohort. Satisfaction was driven primarily by postoperative aesthetic factors—particularly contour quality, scarring, redundant skin, and recurrence, highlighting key targets for surgical optimization and patient counselling.

## Introduction

Gynecomastia, the benign enlargement of male breast tissue, is a common condition affecting up to 65% of men at some point in their lives. It can result from hormonal imbalances, medication side effects, or idiopathic causes, and often leads to significant psychological distress, including embarrassment, reduced self-esteem, and social withdrawal. The physical and emotional impact of gynecomastia frequently motivates patients to seek surgical correction to achieve a more masculine chest contour and improved quality of life [1, 2].

Traditional surgical management has evolved from open excisional techniques, which can leave visible scars and carry higher complication rates, to minimally invasive procedures. Among these, ultrasound-assisted liposuction (UAL) has gained prominence for its ability to efficiently remove both adipose and fibrous glandular tissue while minimizing scarring and promoting skin retraction [3]. UAL is often combined with infra-areolar or periareolar excision to address residual glandular tissue, especially in moderate to severe cases. This combination aims to optimize both functional and aesthetic outcomes, reduce morbidity, and expedite recovery [4].

Patient satisfaction is a critical outcome in gynecomastia surgery, encompassing not only the physical results but also psychological well-being and quality of life. Studies consistently report high satisfaction rates with UAL-based techniques, citing improved chest contour, minimal scarring, and rapid return to daily activities [5]. For example, Hodgson et al. (2005) found that patients rated their overall satisfaction, scar appearance, and chest shape highly after UAL, with average scores above 9 out of 10 and significant improvements in self-confidence [6]. Hasanyn and Saied (2022) reported a 92% success rate and minimal complications in grade III gynecomastia patients treated with UAL and periareolar excision, with most complications being minor and self-limited [7]. Systematic reviews further support these findings, highlighting consistent improvements in quality of life and satisfaction, though they also note variability in how satisfaction is measured across studies [8].

Comparative studies suggest that UAL with infra-areolar excision offers superior aesthetic outcomes and higher satisfaction compared to traditional open excision or liposuction alone, particularly in moderate to severe cases [9–11]. The minimally invasive nature of UAL reduces visible scarring and expedites recovery, which is especially valued by patients concerned about cosmetic results. Complication rates for UAL-based techniques are generally low, with minor issues such as seroma, transient sensory changes, or contour irregularities being the most common [12].

Despite robust evidence supporting UAL-based techniques, there is a notable lack of data from Middle Eastern populations, including Palestine. Regional studies are essential to account for cultural attitudes toward body image, scarring, and healthcare access, which may influence patient satisfaction and expectations [5, 13]. This study aims to assess the satisfaction of gynecomastia patients in Palestine following ultrasound-assisted liposuction with infra-areolar excision, providing region-specific data to inform clinical practice and patient counseling in similar settings. This study is the first to systematically assess patient satisfaction after UAL with infra-areolar excision for gynecomastia in a Palestinian cohort that is expected to provide essential, context-specific evidence to inform both clinical practice and patient counseling in Palestine and similar settings.

## Methodology

### Study design and settings

This was a retrospective cross-sectional study that was conducted to assess patients’ satisfaction after ultrasound-assisted liposuction (UAL) with infra-areolar excision for gynecomastia. The study used the validated Arabic version of the BODY-Q questionnaire, specifically, the chest and nipple modules. Clinical and perioperative data were extracted from medical records, and outcomes were supplemented with patient-reported satisfaction and symptoms collected via an online questionnaire administered at follow-up.

### Study population

The study targeted patients who underwent bilateral surgery between September 2022 and June 2024 at a secondary hospital in Palestine. Inclusion criteria matched those used in similar cross-sectional studies on gynecomastia surgery outcomes, such as age ≥ 16 years, clinical diagnosis of bilateral gynecomastia (Simon grades I–III), and the ability to complete patient-reported outcome measures [13–15]. Patients with previous breast surgery, underlying malignancy, or systemic disease affecting breast tissue were excluded. A total of 133 male patients who underwent UAL with infra-areolar excision for gynecomastia were recruited and asked to fill out an online survey.

### Surgical procedure

All procedures were performed under general anesthesia with the patient in the supine position and the arms abducted. Through a small inferior periareolar (infra-areolar) incision, tumescent solution was infiltrated (approximately 200–500 mL per side, adjusted to breast size and gynecomastia grade). Ultrasound-assisted liposuction (UAL) was then performed using the Ultra-Z system. Emulsification was carried out in a systematic, fan-shaped pattern across the breast (subcutaneous plane), followed by suction-assisted aspiration through a separate ~ 5-mm axillary incision to achieve uniform contouring and feathering at the periphery. Z-mode energy was subsequently applied to promote skin retraction (using manufacturer-recommended settings and treating until a clinically uniform endpoint was achieved).

After liposuction, the inferior periareolar incision was extended to approximately 1.5 cm to allow direct excision of residual glandular tissue bilaterally. Meticulous hemostasis was ensured. Skin was closed with interrupted 3 − 0 Monocryl simple sutures, and sterile dressings were applied. Drains were not routinely. Postoperatively, all patients were instructed to wear a compression chest garment for 4–6 weeks and to avoid heavy lifting and strenuous activity during this period; driving was discouraged for 1–2 weeks, with gradual return to normal activities as tolerated.

### Data collection

Clinical data were obtained retrospectively from the electronic medical records of participating hospitals and were categorized into preoperative, intraoperative, postoperative, and follow-up variables. Preoperative variables included patient demographics and baseline clinical information, such as age, body mass index (BMI), etiology of gynecomastia (classified as physiological, pathological, or drug-induced), and the chief presenting complaint, which ranged from physical enlargement and pain to cosmetic concerns or psychological distress. Additional preoperative data included the presence or absence of chronic illnesses, the use of current medications, and any family history of gynecomastia. The clinical severity of the condition was classified using the Simon grading system (Grades I, IIa, IIb, or III), and the duration of gynecomastia before surgery was recorded as less than 6 months, between 6 and 12 months, or greater than 12 months.

Intraoperative variables focused on surgical details, including the total volume of fat aspirated during ultrasound-assisted liposuction, whether skin excision was performed intraoperatively, and the occurrence of intraoperative bleeding.

Postoperative variables included the presence or absence of complications following surgery. These complications encompassed hematoma, seroma, surgical site infection, asymmetry, contour irregularities, undesirable scarring, adherence or necrosis of the nipple-areola complex, sensory disturbances, persistent residual glandular tissue, the need for revision surgery, persisting pain, recurrence of gynecomastia, and the development of redundant or excess skin.

Postoperative satisfaction was assessed via an online questionnaire administered at follow-up, at varying intervals, and the time since surgery (in months) was recorded as a covariate.

### Patient satisfaction assessment

Patient satisfaction was measured using the validated Arabic version of the BODY-Q questionnaire, specifically the chest and nipple modules. The BODY-Q is a rigorously developed and widely used patient-reported outcome instrument for chest contouring surgery, including gynecomastia, and has demonstrated strong reliability and validity in multiple languages and settings [14, 16–18]. The chest and nipple modules assess satisfaction with chest appearance, nipple-areola complex, and related psychosocial outcomes. The chest module consists of eleven questions; however, ten questions contribute to the total score of 40. The nipple module is composed of five questions, which all contribute to the total score of 20. Raw scores from the BODY-Q scales were summed and converted into Rasch transformed scores (scale of 0–100) using conversion tables obtained from the authors of the BODY-Q.

### Data analysis

Descriptive statistics were used to summarize patient demographics and clinical characteristics (preoperative, intraoperative, and postoperative). Categorical variables are presented as frequency and percentage, and continuous variables are presented as median (interquartile range [IQR]) due to non-normal distributions. Patient satisfaction (BODY-Q chest and nipple modules) was summarized using descriptive statistics and illustrated with bar charts. For inferential analyses, Mann–Whitney U tests were used to compare satisfaction scores between two independent groups, and Kruskal–Wallis tests were used for comparisons across ≥ 3 groups (e.g., follow-up interval and Simon grade). When Kruskal–Wallis tests were significant, pairwise post-hoc comparisons were performed with Bonferroni adjustment. Associations between continuous variables (e.g., BMI, aspirated volume, time since surgery) and satisfaction scores were assessed using Spearman’s rank correlation (ρ). Multivariable linear regression models were constructed to identify independent predictors of chest and nipple satisfaction scores; binary predictors were entered as categorical variables (coded 1 = Yes, 2 = No). A two-sided *p* < 0.05 was considered statistically significant. All analyses were performed using SPSS version 21.

### Ethical considerations

The study protocol was approved by the institutional ethics committee at the Arab American University, Palestine (Reference number: J-2025/A/7/N). All data were anonymized, and patient confidentiality was maintained throughout the study. Informed consent was taken from the participants before starting the questionnaire.

## Results

A total of 133 male patients were included in the analysis. The median age was 28 years (range 24–35), and the median BMI was 26 kg/m² (range 24–31.5) (Table 1). Physiological gynecomastia was the predominant etiology, accounting for 78.9% of cases, while 20.3% were drug induced and only 0.8% were classified as pathological. The most frequent chief complaint was breast enlargement (54.1%), followed by psychological distress (23.3%) and cosmetic concerns (22.6%). Only a small proportion of patients had chronic medical conditions (3.8%) or were on chronic medications (3.0%). A family history of gynecomastia was reported by 17.3% of patients. According to Simon’s classification, 11.3% of patients were grade I, 34.6% grade IIa, 38.3% grade IIb, and 15.8% grade III. The clinical course was long-standing in nearly all cases, with 99.2% reporting symptoms for more than 12 months (Table 1).

**Table 1:** Baseline demographic and clinical characteristics of patients undergoing surgery for gynecomastia

	Median (IQR)
Age	28 (24–35)
BMI	26 (24-31.5)

	Frequency (N)	Percentage %
Etiology		
Pathological	1	0.8
Physiological	105	78.9
Drug induced	27	20.3
Chief complaint		
Enlargement	72	54.1
Cosmetic	30	22.6
Psychological	31	23.3
Chronic diseases		
Yes	5	3.8
No	128	96.2
Chronic medications		
Yes	4	3.0
No	129	97.0
Family history of gynecomastia		
Yes	23	17.3
No	110	82.7
Simon’s Classification		
I	15	11.3
IIa	46	34.6
IIb	51	38.3
III	21	15.8
Course of the disease		
6–12 months	1	0.8
More than 12 months	132	99.2

Intraoperative adverse events were uncommon (Table 2). Clinically relevant bleeding was documented in 2.3% of patients, and additional skin removal was required in only 0.8%. The median aspirated fat volume was 350 mL (range 200–465 mL), reflecting substantial contouring in many cases. Early postoperative complications included hematoma in 5.3% of patients, seroma in 3.0%, and surgical site infection in 0.8%. Aesthetic and contour-related complications were more frequent: 15.8% developed some degree of breast asymmetry, 12.8% had contour irregularity, and 6.0% experienced undesirable scars. Nipple–areola complex (NAC)–related complications were infrequent but clinically relevant: NAC adherence occurred in 4.5%, NAC necrosis in 2.3%, and sensory changes in 3.8% of patients. Residual glandular tissue was detected in 3.0% of cases. Redundant skin was observed in 14.3% of patients, recurrence of gynecomastia in 2.3%, and persistent pain in 0.8%. Overall, 12.0% of patients underwent revision surgery (Table 2).

**Table 2:** Operative findings and postoperative complication profile

Intra-operative complications		
	Frequency (N)	Percentage %
Bleeding		
Yes	3	2.3
No	130	97.7
Skin removal		
Yes	1	0.8
No	132	99.2
Fat-Volume, median (IQR)	350 (200–465)	
Post-operative Complications		
Infection		
Yes	1	0.8
No	132	99.2
Hematoma		
Yes	7	5.3
No	126	94.7
Seroma		
Yes	4	3.0
No	129	97.0
Asymmetry		
Yes	21	15.8
No	112	84.2
Contour irregularity		
Yes	17	12.8
No	116	87.2
Undesirable scar		
Yes	8	6.0
No	125	94.0
Nipple-areola complex adherence		
Yes	6	4.5
No	127	95.5
Nipple-areola complex necrosis		
Yes	3	2.3
No	130	97.7
Sensory changes		
Yes	5	3.8
No	128	96.2
Residual glandular tissue		
Yes	4	3.0
No	129	97.0
Revision		
Yes	16	12.0
No	117	88.0
Persistent pain		
Yes	1	0.8
No	132	99.2
Recurrence		
Yes	3	2.3
No	130	97.7
Redundant skin		
Yes	19	14.3
No	114	85.7

**Fig. 1:**
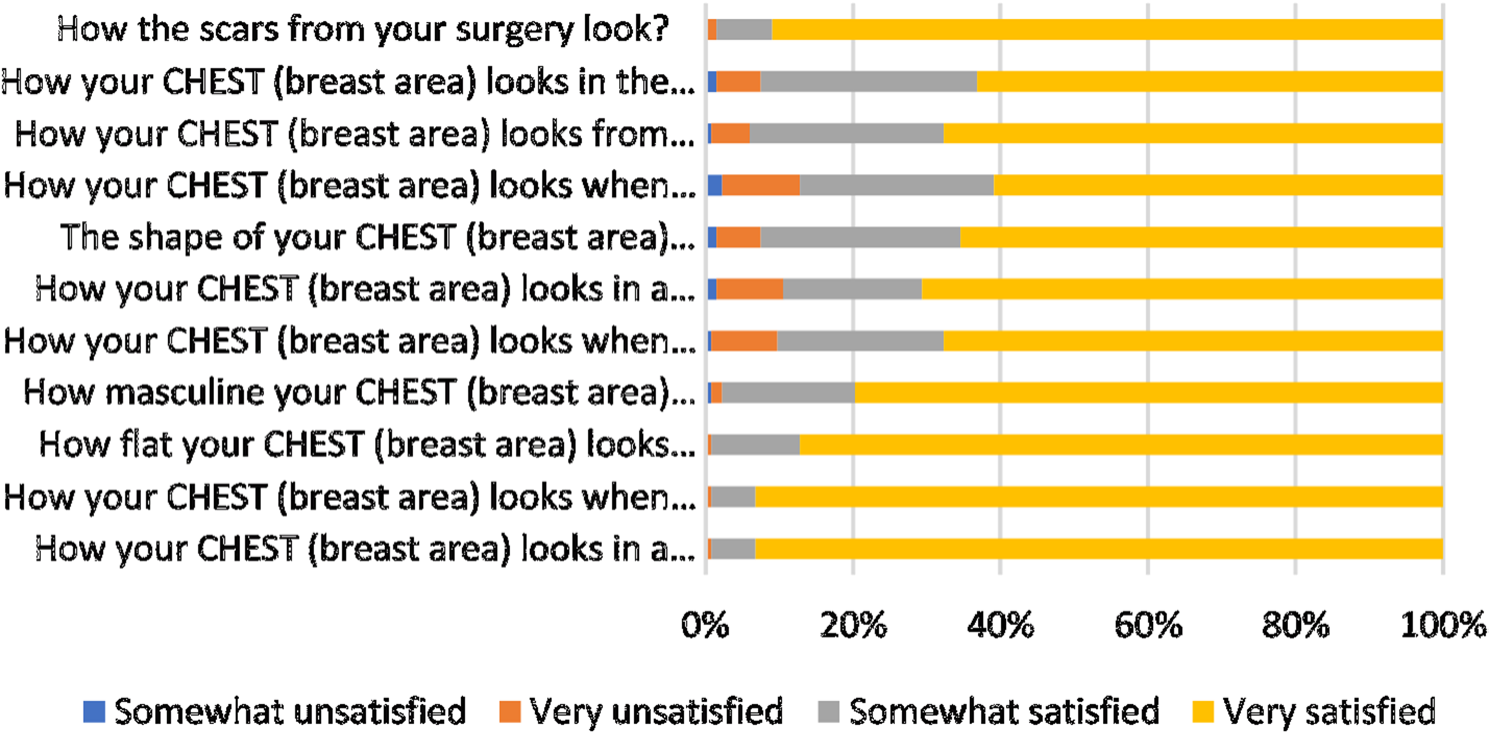
Illustrates patient satisfaction scores derived from the BODY-Q chest module. The X-axis represents the Rasch-transformed satisfaction scores on a scale from 0 to 100, while the Y-axis lists the individual items from the chest module questionnaire. Each item score reflects the level of satisfaction regarding specific aspects of chest appearance and psychosocial perception

**Fig. 2:**
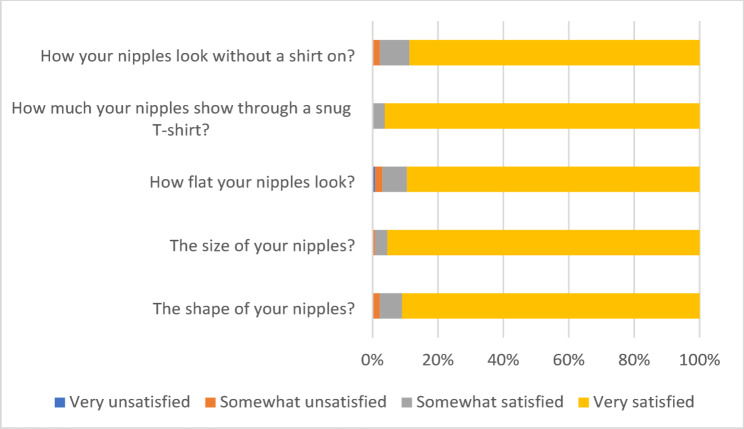
Presents satisfaction ratings from the BODY-Q nipple module, showcasing patient-reported outcomes on the appearance of the nipple-areola complex. The x-axis displays Rasch-transformed scores (0–100), while the y-axis lists the five evaluated items, including nipple shape, size, flatness, and visibility with or without clothing

Despite these complications, overall patient-reported satisfaction with chest contour was high. As shown in Fig. 1, more than 90% of patients were very satisfied with their chest appearance in a loose T-shirt, when lying on their back, and with their scars. High satisfaction was also reported for chest flattening while standing (87.2%), snug T-shirt appearance (70.7%), shirtless appearance (65.0%), and the side view (67.7%) (Fig. 1). With regard to nipple appearance, 91–96% of patients reported high satisfaction with nipple shape, size, flatness, and visibility both with and without clothing (Fig. 2).

When satisfaction was analyzed using Rasch-transformed BODY-Q scores (0–100), median chest satisfaction scores were generally at or near the ceiling in most subgroups (Table 3). Etiology (physiological vs. drug induced) and chief complaint (enlargement, cosmetic, or psychological) did Not significantly affect chest or nipple satisfaction. In contrast, patients with chronic diseases reported significantly lower chest satisfaction (median 61 vs. 100, *p* = 0.023), although nipple satisfaction remained uniformly high in this group (*p* = 0.763). A positive family history of gynecomastia was also associated with lower chest satisfaction (median 79 vs. 100, *p* = 0.030), with a non-significant trend toward lower nipple satisfaction (*p* = 0.083) (Table 3).

**Table 3:** Chest and nipple satisfaction scores (BODY-Q) according to patient characteristics, complications, and surgical outcomes

Name of variable		Chest score-median (IQR)	*P* value		Nipple score-median (IQR)	*P* value
Etiology						
	Physiological	100 (73–100)	0.739	Physiological	100 (100–100)	0.493
	Drug induced	100 (71.5–100)		Drug induced	100 (100–100)	
Chief complain						
	Enlargement	100 (70–100)	0.176	Enlargement	100 (100–100)	0.651
	Cosmetic	100 (83–100)		Cosmetic	100 (100–100)	
	Psychological	76 (70–100)		Psychological	100 (100–100)	
Chronic disease						
	Yes	61 (61–73)	0.023	Yes	100 (100–100)	0.763
	No	100 (73–100)		No	100 (100–100)	
Chronic medication						
	Yes	67 (61-86.5)	0.100	Yes	100 (100–100)	0.411
	No	100 (73–100)		No	100 (100–100)	
Family history of gynecomastia						
	Yes	79 (67–100)	0.030	Yes	100 (95–100)	0.083
	No	100 (76–100)		No	100 (100–100)	
Simon’s Classification						
	I	100 (100–100)	< 0.001	1	100 (100–100)	0.372
	IIa	100 (100–100)		2a	100 (100–100)	
	IIb	87 (70–100)		2b	100 (100–100)	
	III	73 (61–87)		3	100 (100–100)	
Course of disease						
	3.00	100 (73–100)		More than one year	100 (100–100)	
Skin removal						
	No	100 (73–100)	0.094	No	100 (100–100)	0.005
Bleeding						
	Yes	67 (64-83.5)	0.219	Yes	100 (84–100)	0.295
	No	100 (73–100)		No	100 (100–100)	
Infection						
	No	100 (73–100)	0.423	No	100 (100–100)	0.684
Hematoma						
	Yes	100 (64–100)	0.528	Yes	100 (84–100)	0.217
	No	100 (73–100)		No	100 (100–100)	
Seroma						
	Yes	100 (91.5–100)	0.364	Yes	100 (91–100)	0.588
	No	100 (73–100)		No	100 (100–100)	
Asymmetry						
	Yes	73 (64–87)	< 0.001	Yes	100 (100–100)	0.148
	No	100 (79–100)		No	100 (100–100)	
Contour irregularity						
	Yes	70 (54–79)	< 0.001	Yes	100 (82–100)	0.039
	No	100 (77.5–100)		No	100 (100–100)	
Undesirable scar						
	Yes	70 (60–78)	0.004	Yes	82 (56.5–91)	< 0.001
	No	100 (76–100)		No	100 (100–100)	
Nipple–areola complex						
	Yes	100 (79–100)	0.711	Yes	91 (62–100)	0.006
	No	100 (73–100)		No	100 (100–100)	
Nipple–areola necrosis						
	Yes	73 (70-86.5)	0.373	Yes	82 (75–82)	< 0.001
	No	100 (73–100)		No	100 (100–100)	
Sensory changes						
	Yes	67 (61–73)	0.031	Yes	82 (68–82)	< 0.001
	No	100 (73–100)		No	100 (100–100)	
Residual glandular tissue						
	Yes	100 (85–100)	0.536	Yes	100 (100–100)	0.411
	No	100 (73–100)		No	100 (100–100)	
Revision						
	Yes	71.5 (60–100)	0.006	Yes	100 (100–100)	0.982
	No	100 (76–100)		No	100 (100–100)	
Persistent pain						
	No	100 (73–100)	0.062	No	100 (100–100)	0.684
Recurrence						
	Yes	35 (27-45.5)	0.001	Yes	100 (84–100)	0.295
	No	100 (73–100)		No	100 (100–100)	
Redundant skin						
	Yes	70 (60–76)	0.001	Yes	100 (82–100)	0.002
	No	100 (83–100)		No	100 (100–100)	

Chest satisfaction varied significantly with gynecomastia severity according to Simon’s classification. Patients with grade I and grade IIa deformities had median chest scores of 100 (interquartile range [IQR] 100–100), whereas satisfaction declined in grade IIb (median 87) and was lowest in grade III (median 73; *p* < 0.001). In contrast, nipple satisfaction remained uniformly high across all grades, with median scores of 100 in each category and no significant differences (*p* = 0.372). These findings are consistent with the item-level distributions in Figs. 1 and 2, which show high Rasch-transformed scores for both chest and nipple appearance domains (Table 3; Figs. 1 and 2).

The impact of complications on satisfaction was particularly pronounced for chest outcomes (Table 3). Patients who developed breast asymmetry, contour irregularity, undesirable scars, sensory changes, underwent revision surgery, experienced recurrence, or had redundant skin all reported substantially lower chest satisfaction scores. Median chest scores were reduced in the presence of asymmetry (73 vs. 100, *p* < 0.001), contour irregularity (70 vs. 100, *p* < 0.001), undesirable scars (70 vs. 100, *p* = 0.004), and sensory changes (67 vs. 100, *p* = 0.031). Patients requiring revision surgery had lower chest satisfaction (71.5 vs. 100, *p* = 0.006). The greatest decline in chest satisfaction was seen among patients with recurrence (median 35 vs. 100, *p* = 0.001) and those with redundant skin (70 vs. 100, *p* = 0.001) (Table 3).

Nipple satisfaction scores demonstrated a marked ceiling effect, with median values of 100 in almost all subgroups (Table 3). Nevertheless, several complications were associated with statistically significant, albeit more modest, reductions in nipple satisfaction. Contour irregularity (*p* = 0.039), undesirable scars (median 82 vs. 100, *p* < 0.001), NAC adherence (median 91 vs. 100, *p* = 0.006), NAC necrosis (median 82 vs. 100, *p* < 0.001), sensory changes (median 82 vs. 100, *p* < 0.001), and redundant skin (*p* = 0.002) were all associated with lower nipple satisfaction. By contrast, etiology, chief complaint, chronic disease, chronic medications, hematoma, seroma, breast asymmetry, residual glandular tissue, revision surgery, persistent pain, and recurrence did Not significantly influence nipple satisfaction (Table 3).

Table 4 demonstrate that chest satisfaction differed significantly according to the postoperative assessment interval (i.e., the time point at which satisfaction was measured after surgery) (Kruskal–Wallis H [2] = 8.585, *p* = 0.014; *N* = 133). Scores were highest when assessed at 12–18 months (100 [84–100]) and lower at 19–24 months (85 [67–100]) and 25–36 months (87 [67–100]). Bonferroni-adjusted post-hoc testing showed a significant difference only between the 12–18 and 19–24-month assessment groups (*p* = 0.041). Nipple satisfaction did not differ by postoperative assessment interval (*p* = 0.275) and showed a ceiling effect (median 100 [100–100] in all groups).

**Table 4:** Comparison of chest and nipple satisfaction by postoperative follow-up duration

Outcome	12–18 months (*n* = 76)	19–24 months (*n* = 34)	25–36 months (*n* = 23)	*p*-value
Chest satisfaction score	100 (84–100)	85 (67–100)	87 (67–100)	0.014
Nipple satisfaction score	100 (100–100)	100 (100–100)	100 (100–100)	0.275

When patient characteristics and complications were examined across Simon’s Classification, most baseline variables—etiology, chief complaint, chronic medications, presence of chronic disease, and recurrence—did Not differ significantly by grade (Table 5). Family history of gynecomastia showed a borderline association with severity, tending to be more frequent in higher grades (*p* = 0.066). Several complications, however, were clearly related to gynecomastia grade. Contour irregularity was significantly more frequent in grade IIb (25.5%) and also present in grade III (9.5%), compared with very low rates in grades I and IIa (*p* = 0.005). The need for revision surgery increased with deformity grade, from 0% in grade I, to 6.5% in grade IIa, 23.5% in grade IIb, and 4.8% in grade III (*p* = 0.013). Sensory changes were absent in grades I and IIa but occurred in 3.9% of grade IIb and 14.3% of grade III patients (*p* = 0.033). Redundant skin showed the most pronounced gradient, being observed in 52.4% of grade III patients compared with 11.8% in grade IIb, 4.3% in grade IIa, and None in grade I (*p* < 0.001) (Table 5).

**Table 5:** Clinical characteristics and postoperative complications according to gynecomastia classification (Simon’s Classification)

	Class I	Class IIa	Class IIb	Class III	P value
Etiology					0.249
Pathological	0 (0.0%)	0 (0.0%)	0 (0.0%)	1 (4.8%)	
Physiological	11 (73.3%)	34 (73.9%)	44 (86.3%)	16 (76.2%)	
Drug induced	4 (26.7%)	12 (26.1%)	7 (13.7%)	4 (19.0%)	
Chief complain					0.822
Enlargement	10 (66.7%)	25 (54.3%)	27 (52.9%)	10 (47.6%)	
Cosmetic	3 (20.0%)	12 (26.1%)	11 (21.6%)	4 (19.0%)	
Psychological	2 (13.3%)	9 (19.6%)	13 (25.5%)	7 (33.3%)	
Chronic medications					0.280
Yes	0 (0.0%)	1 (2.2%)	1 (2.0%)	2 (9.5%)	
No	15 (100.0%)	45 (97.8%)	50 (98.0%)	19 (90.5%)	
Presence of Chronic diseases					0.422
Yes	0 (0.0%)	1 (2.2%)	2 (3.9%)	2 (9.5%)	
No	15 (100.0%)	45 (97.8%)	49 (96.1%)	19 (90.5%)	
Family history of gynecomastia					0.066
Yes	0 (0.0%)	7 (15.2%)	9 (17.6%)	7 (33.3%)	
No	15 (100.0%)	39 (84.8%)	42 (82.4%)	14 (66.7%)	
Course of the disease					0.113
6–12 months	1 (6.7%)	0 (0.0%)	0 (0.0%)	0 (0.0%)	
More than 12 months	14 (93.3%)	46 (100.0%)	51 (100.0%)	21 (100.0%)	
Skin removal					1.000
Yes	0 (0.0%)	0 (0.0%)	1 (2.0%)	0 (0.0%)	
No	15 (100.0%)	46 (100.0%)	50 (98.0%)	21 (100.0%)	
Wound infection					1.000
Yes	0 (0.0%)	0 (0.0%)	1 (2.0%)	0 (0.0%)	
No	15 (100.0%)	46 (100.0%)	50 (98.0%)	21 (100.0%)	
Breast asymmetry					0.059
Yes	0 (0.0%)	6 (13.0%)	13 (25.5%)	2 (9.5%)	
No	15 (100.0%)	40 (87.0%)	38 (74.5%)	19 (90.5%)	
Contour irregularity					**0.005**
Yes	0 (0.0%)	2 (4.3%)	13 (25.5%)	2 (9.5%)	
No	15 (100.0%)	44 (95.7%)	38 (74.5%)	19 (90.5%)	
Undesirable scar					0.243
Yes	1 (6.7%)	1 (2.2%)	3 (5.9%)	3 (14.3%)	
No	14 (93.3%)	45 (97.8%)	48 (94.1%)	18 (85.7%)	
Nipple areola complex					0.137
Yes	0 (0.0%)	1 (2.2%)	5 (9.8%)	0 (0.0%)	
No	15 (100.0%)	45 (97.8%)	46 (90.2%)	21 (100.0%)	
Nipple areola necrosis					0.098
Yes	0 (0.0%)	0 (0.0%)	1 (2.0%)	2 (9.5%)	
No	15 (100.0%)	46 (100.0%)	50 (98.0%)	19 (90.5%)	
Seroma					0.241
Yes	1 (6.7%)	0 (0.0%)	3 (5.9%)	0 (0.0%)	
No	14 (93.3%)	46 (100.0%)	48 (94.1%)	21 (100.0%)	
Recurrence					0.489
Yes	0 (0.0%)	0 (0.0%)	2 (3.9%)	1 (4.8%)	
No	15 (100.0%)	46 (100.0%)	49 (96.1%)	20 (95.2%)	
Persistent pain					1.000
Yes	0 (0.0%)	0 (0.0%)	1 (2.0%)	0 (0.0%)	
No	15 (100.0%)	46 (100.0%)	50 (98.0%)	21 (100.0%)	
Revision					**0.013**
Yes	0 (0.0%)	3 (6.5%)	12 (23.5%)	1 (4.8%)	
No	15 (100.0%)	43 (93.5%)	39 (76.5%)	20 (95.2%)	
Residual glandular tissue					0.753
Yes	0 (0.0%)	2 (4.3%)	2 (3.9%)	0 (0.0%)	
No	15 (100.0%)	44 (95.7%)	49 (96.1%)	21 (100.0%)	
Sensory changes					**0.033**
Yes	0 (0.0%)	0 (0.0%)	2 (3.9%)	3 (14.3%)	
No	15 (100.0%)	46 (100.0%)	49 (96.1%)	18 (85.7%)	
Hematoma					0.249
Yes	0 (0.0%)	2 (4.3%)	5 (9.8%)	0 (0.0%)	
No	15 (100.0%)	44 (95.7%)	46 (90.2%)	21 (100.0%)	
Bleeding					0.192
Yes	0 (0.0%)	0 (0.0%)	3 (5.9%)	0 (0.0%)	
No	15 (100.0%)	46 (100.0%)	48 (94.1%)	21 (100.0%)	
Redundant skin					< 0.001
Yes	0 (0.0%)	2 (4.3%)	6 (11.8%)	11 (52.4%)	
No	15 (100.0%)	44 (95.7%)	45 (88.2%)	10 (47.6%)	

Correlation analysis further clarified the determinants of satisfaction (Table 6). Chest satisfaction scores showed a moderate negative correlation with BMI (Spearman *r* = − 0.467, *p* < 0.001), indicating that higher BMI was associated with lower chest satisfaction. There were weaker but still significant negative correlations between chest satisfaction and age (*r* = − 0.185, *p* = 0.033), fat volume removed (*r* = − 0.417, *p* < 0.001), and time since surgery (*r* = − 0.284, *p* < 0.001). Thus, heavier and older patients, those requiring larger volumes of fat removal, and those with longer follow-up tended to report lower chest satisfaction. In contrast, nipple satisfaction scores were largely independent of these variables; only fat volume removed demonstrated a weak but significant negative correlation with nipple satisfaction (*r* = − 0.192, *p* = 0.027). Importantly, chest and nipple satisfaction scores were positively correlated with each other (*r* = 0.358, *p* < 0.001), suggesting that patients who were more satisfied with chest contour were also more satisfied with nipple appearance (Table 6).

**Table 6:** Correlation between satisfaction scores and BMI, age, fat volume, time since surgery

Variable	Chest satisfaction score		Nipple satisfaction score	
	Spearman correlation coefficient	*P* value	Spearman correlation coefficient	*P* value
BMI	-0.467	< 0.001	-0.128	0.141
Age	-0.185	0.033	0.075	0.394
Fat volume	-0.417	< 0.001	-0.192	0.027
Time since surgery	-0.284	< 0.001	-0.078	0.373
Nipple satisfaction score	0.358	< 0.001		
Chest satisfaction score			0.358	< 0.001

In the multivariable linear regression model for chest satisfaction (BODY-Q chest module), demographic factors (age, BMI), time since surgery, presence of chronic disease, family history, gynecomastia grade, fat volume removed, sensory changes, and revision surgery were Not independently associated with chest satisfaction after adjustment (Table 7). Instead, several aesthetic and structural factors remained significant predictors. Contour irregularity (*p* = 0.013), undesirable scars (*p* = 0.019), redundant skin (*p* < 0.001), and recurrence (*p* < 0.001) were all independently associated with lower chest satisfaction scores, underscoring that postoperative contour quality, scar appearance, management of excess skin, and long-term stability of the result are key determinants of perceived success (Table 7).

**Table 7:** Multiple linear regression model for chest satisfaction score (BODY-Q Chest module)

Predictor	B (unstandardized)	SE(B)	β (standardized)	t	*p*-value	95% CI for B
Age (years)	−0.139	0.137	−0.062	−1.01	0.313	−0.410 to 0.133
BMI (kg/m²)	−0.410	0.327	−0.115	−1.25	0.213	−1.057 to 0.238
Time since surgery	−0.277	0.186	−0.101	−1.49	0.140	−0.646 to 0.092
Chronic disease	8.411	5.677	0.090	1.48	0.141	−2.83 to 19.65
Family history of gynecomastia	3.398	2.788	0.072	1.22	0.225	−2.12 to 8.92
Simon’s Classification	−0.950	2.437	−0.047	−0.39	0.697	−5.78 to 3.88
Fat volume removed (mL)	−0.010	0.013	−0.086	−0.74	0.463	−0.035 to 0.016
Asymmetry	6.913	3.898	0.141	1.77	0.079	−0.81 to 14.63
Contour irregularity	9.764	3.850	0.183	2.54	0.013	2.14 to 17.39
Undesirable scar	17.703	7.434	0.236	2.38	0.019	2.98 to 32.43
Redundant skin	15.445	3.351	0.303	4.61	< 0.001	8.81 to 22.08
Recurrence	50.523	7.287	0.420	6.93	< 0.001	36.09 to 64.95
Sensory changes	−11.231	9.002	−0.120	−1.25	0.215	−29.06 to 6.59
Revision surgery	0.638	3.894	0.012	0.16	0.870	−7.07 to 8.35

Binary predictors were coded as 1 = Yes and 2 = No. Therefore, a positive β indicates higher satisfaction when the condition/complication is absent (No vs. Yes)

For nipple satisfaction (BODY-Q nipple module), the multivariable model similarly highlighted aesthetic factors as the primary drivers of patient-reported outcomes (Table 8). Age, BMI, gynecomastia grade, fat volume removed, time since surgery, sensory changes, and NAC necrosis were Not significant independent predictors. In contrast, contour irregularity (*p* = 0.014), undesirable scars (*p* < 0.001), redundant skin (*p* = 0.005), and NAC “complex”/adherence (*p* = 0.001) were all independently associated with lower nipple satisfaction. These findings indicate that, beyond baseline characteristics and deformity grade, the quality of contour refinement, scarring, management of redundant skin, and preservation of NAC mobility are the main determinants of how patients rate the aesthetic outcome of the nipple–areola complex (Table 8).

**Table 8:** Multiple linear regression model for nipple satisfaction score (BODY-Q nipple module)

Predictor	B (unstandardized)	SE(B)	β (standardized)	t	*p*-value	95% CI for B
Age (years)	0.17	0.10	0.13	1.70	0.091	−0.03 to 0.37
BMI (kg/m²)	0.08	0.24	0.04	0.35	0.727	−0.39 to 0.55
Simon’s Classification	1.50	1.74	0.12	0.86	0.392	−1.95 to 4.94
Fat volume removed (mL)	−0.01	0.01	−0.16	−1.12	0.266	−0.03 to 0.01
Contour irregularity	5.89	2.36	0.19	2.50	0.014	1.22 to 10.56
Undesirable scar	20.47	5.52	0.46	3.71	< 0.001	9.54 to 31.40
Redundant skin	6.94	2.45	0.23	2.84	0.005	2.09 to 11.79
Sensory changes	5.54	8.31	0.10	0.67	0.506	−10.91 to 21.99
Time since surgery	−0.03	0.14	−0.02	−0.24	0.808	−0.30 to 0.24
NAC “complex”/adherence	12.38	3.80	0.24	3.26	0.001	4.87 to 19.90
NAC necrosis	−5.74	8.00	−0.08	−0.72	0.475	−21.59 to 10.11

Binary predictors were coded as 1 = Yes and 2 = No. Therefore, a positive β indicates higher satisfaction when the condition/complication is absent (No vs. Yes)

## Discussion

This study evaluated patient-reported satisfaction following ultrasound-assisted liposuction (UAL) with infra-areolar glandular excision for gynecomastia in a Middle Eastern population. The results demonstrated high levels of satisfaction in both the BODY-Q chest and nipple domains, with low complication rates overall. Satisfaction was strongly influenced by postoperative aesthetic outcomes—particularly contour regularity, scar quality, redundant skin, and recurrence—rather than demographic or operative variables. These findings align closely with emerging global evidence supporting UAL-based techniques as safe, effective, and aesthetically superior options for gynecomastia correction.

The high satisfaction levels observed in this cohort corroborate previous reports indicating that UAL provides excellent cosmetic outcomes with minimal morbidity. Recent studies have highlighted UAL’s ability to achieve smooth chest contours while minimizing visible scarring and downtime compared to traditional open excision methods [10, 11]. In a prospective pilot by Gelidan and Mortada (2025), the combination of short-scar excision with UAL yielded uniformly high satisfaction rates and negligible major complications—findings closely paralleling those of the current study [10]. Similarly, Bailey and Guenther (2016) reported that the pull-through technique with UAL effectively treated all gynecomastia grades with a near-zero revision rate and excellent contour preservation.

Our results also align with Ouf and Kishk (2024), who found that VASER-assisted liposuction—an ultrasound-based modality—significantly improved aesthetic outcomes and skin firmness while minimizing redundancy and the need for revision surgery [12]. Comparable findings were noted by Eyuboğlu et al. (2024), who showed that extensive ultrasonic liposuction without glandular excision achieved a complication rate below 9%, substantially lower than conventional excisional techniques [5]. Together, these data affirm UAL’s capacity to balance effective tissue removal with aesthetic refinement.

The current study’s regression analyses underscored that postoperative contour irregularity, undesirable scarring, redundant skin, and recurrence were independent predictors of reduced satisfaction—both in the chest and nipple domains. With respect to asymmetry, the observed rate of postoperative asymmetry (15.8%) in our series, while within the acceptable range reported in the literature, deserves particular attention. Asymmetry after gynecomastia surgery is often multifactorial and can arise from uneven liposuction distribution, residual glandular tissue, or asymmetric skin retraction across the chest wall. Technical aspects such as over-resection on one side, insufficient glandular excision, and the surgeon’s learning curve in combining ultrasound-assisted liposuction (UAL) with open excision are recognized contributors [19]. Consistent with previous findings, contour irregularity and asymmetry were key predictors of reduced satisfaction in our regression model, underscoring the importance of achieving uniform tissue removal and maintaining balanced feathering during aspiration [15]. This aligns with the classification framework by Rohrich et al., which emphasizes that residual glandular density and uneven aspiration planes are the leading causes of postoperative asymmetry [1].

To prevent contour irregularities and visible dog-ears, meticulous peripheral feathering was routinely performed, and postoperative compression garments were strictly used. These measures are consistent with recommendations by Rohrich et al. (2003) and Alhotan & Salati (2025), who emphasized that even suction planes and prolonged compression optimize skin redraping and contour uniformity [1, 14]. This pattern mirrors that of Abdelrahman and Steinvall (2018), who reported that contour smoothness and scar quality were the strongest determinants of postoperative satisfaction in glandular liposculpture for grades I–II gynecomastia [13]. Similarly, in a large comparative study, Akhtar and Eitezaz (2019) found no major difference in satisfaction between open excision and minimally invasive arthroscopic-assisted liposuction but noted that contour irregularity was the most common cause of patient dissatisfaction [15].

Redundant skin and nipple–areola complex (NAC) adherence, both found to independently reduce satisfaction in our series, are frequently reported challenges in higher Simon grades. In a multicenter analysis, Alhotan and Salati (2025) emphasized that addressing skin redundancy and preserving NAC pliability are critical for patient-perceived aesthetic success [14]. Their findings—85% patient satisfaction and minimal major complications—underscore the shared importance of scar concealment and natural chest contouring. The positive correlation between chest and nipple satisfaction observed in our study further reinforces that aesthetic harmony across these regions is vital to perceived success, echoing previous BODY-Q–based analyses [16].

For patients with grade III gynecomastia in our cohort, skin redundancy represented a particular aesthetic challenge. Routine skin excision was deliberately avoided to minimize visible scarring; instead, we relied on the intrinsic skin-tightening effect of ultrasound energy (Z-mode) and the use of postoperative compression garments for 4–6 weeks to facilitate dermal retraction and uniform chest contour. Previous research has demonstrated that ultrasound-based modalities such as VASER-assisted liposuction can enhance skin firmness and reduce the need for additional excisional procedures [12]. Similarly, Eyuboğlu (2024) showed that extensive ultrasonic liposuction alone can achieve excellent dermal contraction without formal skin excision [5]. These findings are consistent with Ratnam (2009), who demonstrated that ultrasound-assisted lipoplasty with subdermal stimulation significantly improves skin retraction in gynecomastia patients [18], and with Santoli (2001), who reported similar tightening effects in breast tissue during ultrasound-assisted mastopexy [17]. Troell (2022) further confirmed that UAL achieves superior skin firmness and contour definition compared with power-assisted techniques [20]. Collectively, these studies reinforce that careful ultrasound energy delivery, compression garment adherence, and peripheral feathering can effectively manage moderate-to-severe redundancy without formal skin excision.

Interestingly, neither age, BMI, nor operative fat volume independently predicted satisfaction after adjustment, though BMI correlated modestly with lower unadjusted chest satisfaction. Similar findings were reported by Rahmahi (2020), who found that outcomes and satisfaction were independent of BMI or age but instead were tied to technical precision and postoperative contour regularity [21]. This supports the interpretation that psychosocial satisfaction after gynecomastia correction is more dependent on visual and tactile results than on baseline characteristics. In higher-grade cases, redundant skin and fibrosis—not demographic variables—tend to dictate satisfaction outcomes [5, 22].

When compared with other minimally invasive techniques, such as vacuum-assisted mastectomy combined with power-assisted liposuction (PAL), ultrasound-assisted methods appear to offer distinct aesthetic advantages. Wang et al. reported that vacuum-assisted PAL achieved low complication rates and excellent contour restoration for mixed-type gynecomastia [23]. However, recent comparative studies suggest that UAL’s tissue-selective emulsification allows smoother contouring and less mechanical trauma to surrounding tissues. Eyuboğlu (2024) found that extensive ultrasonic liposuction achieved superior cosmetic outcomes and avoided periareolar scars compared with open excision techniques [5]. Likewise, Gupta and Bansal (2025) demonstrated that combining PAL with UAL through a lateral incision produced excellent contour uniformity and nearly invisible scars [24]. Brown and Chang (2015) similarly highlighted that ultrasound-assisted modalities provide greater precision in fat-gland emulsification and reduced asymmetry compared with purely mechanical systems [25]. Furthermore, a recent systematic meta-analysis by Ahmed and Shraim (2025) confirmed that UAL achieves significantly lower rates of contour irregularities and asymmetry compared with PAL and suction-assisted techniques [26]. Collectively, these findings reinforce that ultrasound-assisted liposuction can yield smoother chest definition and superior scar quality relative to other power-based methods.

This study adds region-specific evidence from Palestine, where cultural perceptions of scarring and body image may uniquely influence satisfaction. The high satisfaction rates observed suggest that UAL with infra-areolar excision effectively meets aesthetic and psychosocial expectations within this context. Given that regional studies in the Middle East remain scarce, these findings contribute valuable insights into patient-centered outcomes and surgical counseling strategies.

The retrospective design and reliance on self-reported satisfaction introduce potential biases related to recall and follow-up duration. Nonetheless, the use of the validated Arabic BODY-Q instrument enhances comparability and reliability. Future multicenter prospective studies with longer follow-up are warranted to assess durability of satisfaction and recurrence, particularly across diverse cultural and clinical settings.

## Conclusion

Ultrasound-assisted liposuction with infra-areolar excision provided high patient-reported satisfaction with generally low complication rates in this Palestinian cohort. While baseline characteristics and follow-up interval were not independent drivers of outcomes, satisfaction was strongly determined by postoperative aesthetic and structural factors. In particular, contour irregularity, undesirable scarring, redundant skin, and recurrence were the key independent predictors of lower chest satisfaction, and similar aesthetic factors—along with nipple–areola complex adherence—were the primary drivers of nipple satisfaction. These findings emphasize that optimizing contour refinement, scar quality, management of skin redundancy, and long-term stability of correction are central to maximizing patient-reported success, especially in higher-grade.

## Data Availability

The datasets used and/or analyzed during the current study are available from the corresponding author upon reasonable request.
